# Risk of dementia in patients with primary insomnia: a nationwide population-based case-control study

**DOI:** 10.1186/s12888-018-1623-0

**Published:** 2018-02-07

**Authors:** Chao-Ming Hung, Ying-Chun Li, Han-Jung Chen, Kang Lu, Cheng-Loong Liang, Po-Chou Liliang, Yu-Duan Tsai, Kuo-Wei Wang

**Affiliations:** 1 Department of General Surgery, E-Da Cancer Hospital, I-Shou University, Kaohsiung, Taiwan; 2 Institute of Health Care Management, National Sun Yat-Sen University, Kaohsiung, Taiwan; 3 Department of Neurosurgery, E-Da Hospital, I-Shou University, Kaohsiung, Taiwan; 4 Department of Neurosurgery, E-Da Cancer Hospital, I-Shou University, Kaohsiung, Taiwan

**Keywords:** Insomnia, Sleep disorder, Dementia, Neurodegeneration, Epidemiology

## Abstract

**Background:**

To investigate the association between primary insomnia and dementia using a Taiwanese population-based database.

**Methods:**

This case-control study involved a subset of Taiwan’s National Health Insurance Research Database of reimbursement claims. We included 51,734 patients who were diagnosed with primary insomnia from 2002 to 2004 as the test group and 258,715 nonprimary insomnia participants aged 20 years or older as the reference group. We excluded patients under 20 and those with depression, post-traumatic stress disorder, and/or sleep disorders caused by organic lesion(s), drugs, or alcohol. We used a Cox proportional hazards model to assess the primary insomnia on the risk of developing dementia after adjusting for sociodemographic characteristics and comorbidities.

**Results:**

The primary insomnia cohort had a higher prevalence of diabetes, dyslipidemia, hypertension, coronary heart disease, chronic liver disease, and chronic kidney disease at baseline. After adjusting for select comorbidities, primary insomnia remained a significant predisposing factor for developing dementia, and was associated with a 2.14-fold (95% confidence interval, 2.01–2.29) increase in dementia risk. We also found a higher risk of dementia in younger patients.

**Conclusions:**

Taiwanese patients with primary insomnia, especially those under 40, had a higher risk of developing dementia than those without primary insomnia.

## Background

Insomnia is defined as difficulty in initiating sleep, maintaining sleep, and/or struggling with frequent early morning awakenings. Difficulty in sleeping significantly interfering with daily functioning is an important factor considered before diagnosing a patient with insomnia [1]. Primary insomnia is sleeplessness that is not attributable to a medical, psychiatric, or environmental cause. The predominant symptom is difficulty initiating or maintaining sleep, or suffering from nonrestorative sleep, for at least 1 month. The sleep disturbance causes clinically significant distress or impairment in social, occupational, or other important areas of functioning [2]. Primary insomnia is also associated with hypertension, diabetes, myocardial infarction, stroke, and transient ischemic attack [3, 4]. Although an association between sleep and neurological disorders is widely acknowledged [5, 6], most studies of this association have focused on sleep apnea [7, 8]. Few studies have investigated the relationship between primary insomnia and the prevalence of neurological diseases.

Dementia is a condition involving a significant decrease in cognitive abilities, including memory deficits, mood changes, and problems in communication and reasoning. Approximately 1% of the 35 million people worldwide aged 65 to 69 years are affected by this condition [9], and its prevalence increases with age of the population. The percentage of the Taiwanese population aged 65 and above has increased over the past 27 years from 4.1% in 1980 to 10.2% in 2007 [10]. In 2007, the estimated average life expectancy was 81 and 75 years for women and men, respectively. Therefore, dementia is one of the biggest threats to the health of the Taiwanese population.

In this study, we investigated whether primary insomnia was associated with an increased risk of dementia using the Taiwan Longitudinal Health Insurance Database (LHID 2000). In addition, we investigated whether this association was moderated by age.

## Methods

### Database

This study consisted of a nationwide population-based case-control design. The data were retrieved from the Longitudinal Health Insurance Database (LHID 2000) of Taiwan’s National Health Institute Research Database (NHIRD) [11, 12]. This program is a universal health insurance program that was launched in March 1995 in Taiwan. LHID 2000, which is made available to scientists in Taiwan for research purposes, contains comprehensive healthcare data for 1 million people who were randomly selected from original insurance claim data and the registration files of all beneficiaries, representing more than 99% of the 23.37 million people living in Taiwan from 1996 to 2010. The study was approved by the National Health Research Institutes, Taiwan. The database information was anonymized.

### Study sample

**Fig. 1:**
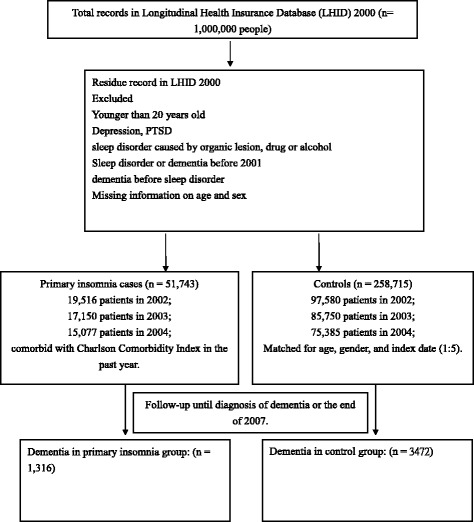
Flow chart describing the study population

For the study cohort, we were interested in determining the effects of primary insomnia on the development of dementia. The diagnoses were based on the International Classification of Diseases, Ninth Revision, Clinical Modification (ICD-9-CM). Initially, we included patients who had visited ambulatory care centers for the treatment of primary insomnia from 2002 to 2004 (*n* = 51,743) [307.4 (specific disorders of sleep of nonorganic origin), 780.5 (sleep disturbance), 780.50 (sleep disturbance, unspecified), 780.52 (insomnia, unspecified), 780.54 (hypersomnia, unspecified), 780.55 (disruptions of the 24-h sleep-wake cycle, unspecified), 780.56 (dysfunctions associated with sleep stages or arousal from sleep), 780.58 (sleep-related movement disorder, unspecified), and 780.59 (sleep disturbance, other)] [13]. Patients were diagnosed with a sleep disorder when at least two ambulatory care claims were recorded [14, 15]. We excluded patients younger than 20 years of age and those with depression, post-traumatic stress disorder, and sleep disorders caused by organic lesion(s), drugs or alcohol [291.82 (alcohol-induced sleep disorders), 292.85 (drug-induced sleep disorders], 309.1 (prolonged depressive reaction), 309.81 (post-traumatic stress disorder), 327.0 (organic disorders of initiating and maintaining sleep), 327.4 (organic parasomnia), 327.5 (organic sleep-related movement disorders), and 327.8 (other organic sleep disorders)]. Dementia is not a specific disease. It is a descriptive term for a collection of symptoms that can be caused by several disorders that affect the brain. People with dementia have significantly impaired intellectual functioning that interferes with normal activities and relationships. They also lose their ability to solve problems and maintain emotional control, and they may experience personality changes and behavioral problems, such as agitation, delusions, and hallucinations [16]. In addition, patients who were diagnosed with dementia [290.0 (senile dementia, uncomplicated), 290.1 (presenile dementia), 290.2 (senile dementia with delusional or depressive features), 290.3 (senile dementia with delirium), 290.4 (arteriosclerotic dementia), 294.1 (dementia in conditions classified elsewhere), 331.0 (Alzheimer’s disease), 331.1 (Pick disease), and 331.2 (senile degeneration of the brain)] prior to the index use of health care facilities were identified and excluded from the study. A total of 51,743 primary insomnia patients were included in this study. The study flow chart is shown in Fig. 1.

The comparison cohort (5 comparison subjects for every patient with sleep disorders) was randomly selected from the remaining age- and sex-matched insured controls that used the health service during the same period. A total of 258,715 controls were enrolled in this study, none of whom had been diagnosed with dementia before their initial clinic visits.

### Main outcome measures

The primary endpoint of this study was the diagnosis of dementia during hospitalization. Dementia diagnosis in the follow-up years was based on the presence of ICD-9-CM codes 290.0, 290.1, 290.2, 290.3, 290.4, 294.1, 331.0, 331.1, and 331.2 [11, 12]. Each patient was followed for a 3-year period after their sleep disorder diagnosis to identify those patients who subsequently developed dementia. We also explored the relationship between primary insomnia and dementia in different age groups.

### Statistical analysis

Our study used Pearson’s χ^2^ tests to examine the differences in sociodemographic characteristics among patients with and without primary insomnia and select comorbid medical disorders. Selected comorbidities that occurred either in the inpatient setting or in two or more ambulatory care claims and that were recorded 1 year before the index ambulatory care visit were considered in our study. We used the Kaplan–Meier method and log-rank test to estimate the 3-year dementia-free rate and compared the risk of dementia for these two cohorts. In addition, a stratified Cox proportional hazard regression (HR; stratified by age group: 20–39, 40–59, 60–74, and ≥75) was conducted to calculate the 3-year dementia-free rate between patients with and without primary insomnia. The HR values and 95% confidence intervals were computed with a significance level of 0.05. All statistical analyses were conducted using SAS 9.2 statistical software (SAS Institute, Inc., Cary, NC, USA).

## Results

Table 1 shows the sociodemographic characteristics and baseline comorbidity statuses of the patients in the primary insomnia and non-primary insomnia cohorts. A total of 51,743 patients who were diagnosed with primary insomnia matched the inclusion criteria; 258,715 patients were included in the comparison cohort. Patients with primary insomnia were more likely to have diabetes (8.25% vs. 6.47%, *p* < 0.0001), dyslipidemia (8.63% vs. 6.05%, *p* < 0.0001), hypertension (18.43% vs. 13.55%, *p* < 0.0001), coronary heart disease (7.35% vs. 4.57%, *p* < 0.0001), chronic liver disease (10.82% vs. 6.43%, *p* < 0.0001), and chronic kidney disease (3.16% vs. 1.96%, *p* < 0.0001).

**Table 1:** Comparison of the Demographic Characteristics and Comorbidities between Patients with and without Primary insomnia

	Patients With primary insomnia		Comparison Patients		
	(*N* = 51,743)		(*N* = 258,715)		
Characteristic	*N*	%	*N*	%	*P*
Sex					1.0000a
Male	20,878	40.35	104,390	40.35	
Female	30,865	59.65	154,325	59.65	
Age, mean ± SD	47.39 ± 15.69		47.39 ± 15.69		1.0000b
Year					1.0000a
≦39	17,637	34.09	88,185	34.09	
40–59	22,081	42.67	110,405	42.67	
60–74	8890	17.18	44,450	17.18	
≥ 75	3135	6.06	15,675	6.06	
Geographic region					<.0001a
Northern	22,354	43.20	123,645	47.79	
Central	14,241	27.52	53,729	20.77	
Southern	12,622	24.39	68,637	26.53	
Eastern	1194	2.31	6362	2.46	
Missing	1332	2.57	6342	2.45	
Comorbidities					
diabetes	4267	8.25	16,750	6.47	<.0001a
dyslipidemia	4465	8.63	15,656	6.05	<.0001a
hypertension	9538	18.43	35,067	13.55	<.0001a
coronary heart disease	3803	7.35	11,824	4.57	<.0001a
chronic liver disease	5597	10.82	16,630	6.43	<.0001a
chronic kidney disease	1636	3.16	5069	1.96	<.0001a

*SD* standard deviation; *a* chi-square test; *b* t-test

Table 2 shows the prevalence of dementia in the study subjects. During the 3-year follow-up period, 1316 patients with primary insomnia (2.54% of the study cohort) and 3472 patients with non-primary insomnia (1.34% of the comparison cohort) developed dementia. Compared with patients without primary insomnia, patients with primary insomnia had a higher risk of developing dementia (HR = 2.17; 95% CI, 2.04–2.32; *p* < 0.05). After adjusting for geographic location, residential area, and select premorbid comorbidities, primary insomnia remained a significant predisposing factor for developing dementia, and was associated with a 2.14-fold (95% CI, 2.01–2.29) increase in dementia risk.

**Table 2:** Dementia risk among sampled patients during the 3-year follow-up period after index healthcare utilization

Type of sleep disorder	n	Persons-years	dementia case	Unadjusted		Adjusted	
				HR	95% CI	HR	95% CI
No insomnia	258,715	1,303,464.02	3472	1.00	(reference)	1.00	(reference)
Primary insomnia	51,745	233,853.23	1316	2.17*	(2.04-2.32)	2.14*	(2.01-2.29)

*CI* confidence interval

*p < 0.05 The Hazard Ratio (HR) was calculated by using the stratified Cox proportional regression method (stratified by sex, age group, and year of index healthcare use) during the 3-year follow-up period. Adjustments were made for the following selected comorbidities in the patients: diabetes, hyperlipidemia, hypertension, coronary heart disease, chronic liver disease, and chronic kidney disease

Table 3 shows the relative risk of dementia according to age. In the stratified Cox proportional HR, the dementia risk yielded the following results for the 3-year period: in those aged 20–39 years, the HR of patients with primary insomnia was 4.88 (95% CI, 2.98–7.99; *p* < 0.05) compared with patients with non-primary insomnia; in subjects aged 40–59, the HR was 3.56 (95% CI, 2.91–4.36; *p* < 0.05); and, in the 60–74 age group, the HR was 2.21 (95% CI, 2.00–2.44; *p* < 0.05). The HR for those older than 75 years was 1.96 (95% CI, 1.79–2.15; *p* < 0.05). Furthermore, we found that the adjusted HR for dementia within the 3-year period in patients with primary insomnia aged 20–39 years was 4.77 (95% CI, 2.92–7.84; *p* < 0.05), aged 40–59 years was 3.24 (95% CI, 2.91–4.36; *p* < 0.05), the corresponding value in patients aged 60–74 years was 2.12 (95% CI, 1.92–2.34; *p* < 0.05), and in patients older than 75 years, the adjusted HR value was 1.95 (95% CI, 1.77–2.14; *p* < 0.05).

**Table 3:** Crude and adjusted Hazard Ratios (HRs) for dementia among sampled patients of different age groups during the 3-year follow-up period after index healthcare utilization

	n	dementia case	Unadjusted		Adjusted	
			HR	95% CI	HR	95% CI
20–39						
No insomnia	88,185	34	1.00	(reference)	1.00	(reference)
Primary insomnia	17,637	30	4.88*	(2.98- 7.99)	4.77*	(2.90-7.84)
40–59						
No insomnia	110,405	253	1.00	(reference)	1.00	(reference)
Primary insomnia	22,081	155	3.56*	(2.91- 4.36)	3.24*	(2.64-3.97)
60–74						
No insomnia	44,450	1392	1.00	(reference)	1.00	(reference)
Primary insomnia	8890	533	2.21*	(2.00- 2.44)	2.12*	(1.92-2.34)
≥ 75						
No insomnia	15,675	1793	1.00	(reference)	1.00	(reference)
Primary insomnia	3135	598	1.96*	(1.79- 2.15)	1.95*	(1.77-2.14)

*p < 0.05 The HR was calculated by using the stratified Cox proportional regression method (stratified by sex, age group, and the year of index healthcare use) during the 3-year follow-up period. Adjustments were made for the following selected comorbidities in the patients: diabetes, hyperlipidemia, hypertension, coronary heart disease, chronic liver disease, and chronic kidney disease

## Discussion

In the present study, patients with primary insomnia showed an increased prevalence of dementia risk factors, such as hypertension, diabetes, hyperlipidemia, and coronary heart disease. Our findings were concurrent with meta-analyses and community-based reports showing that primary insomnia is associated with an increased prevalence of hypertension, diabetes, myocardial infarction, stroke, and transient ischemic attack [17]. Patients with primary insomnia have a greater risk of being diagnosed with dementia. During the 3-year follow up, 2.54% of patients with primary insomnia (1316 patients) compared with 1.34% of patients without primary insomnia (3472 patients) were diagnosed with dementia. After adjusting for age, sex, region of residence, and selected comorbidities, a primary insomnia diagnosis was independently associated with a 2.17-fold higher risk of subsequent development of dementia. When the results were further stratified according to sex and other covariates were controlled, the adjusted HR was 2.14-fold (95% CI, 2.01–2.29). Younger patients with primary insomnia had a higher risk of developing dementia.

The mechanism underlying the association between primary insomnia and dementia is unclear. Sleep disturbances are common in the general population. A recent study conducted in older adults reported that insomnia was associated with a significantly increased risk of all-cause dementia [18]. Another recent study also demonstrated that sleep disturbances can enhance the risk of developing dementia, and insomnia may increase the risk of Alzheimer’s disease. These findings elucidate the influence of sleep disturbances on the incidence of dementia, especially in older adults [19]. In the US, 35–40% of the adult population is reported to suffer from sleep disorders. There is increasing evidence from large screening surveys showing that primary insomnia is associated with hypertension, diabetes, myocardial infarction, stroke, and transient ischemic attack [20–24], which suggests that sleep disorders should be considered a new cardiovascular risk factor. Because diabetes, hypertension, myocardial infarction, and stroke are known risk factors for dementia, these findings suggest that patients with primary insomnia have higher cognitive dysfunction [11, 12].

Several recently published studies support the results of the present study. A study by Monlar et al. showed that restless legs syndrome was associated with an 88% increase in mortality risk, and the risk of coronary heart disease and stroke increased almost four times [25]. Stroke and coronary heart disease are risk factors for dementia [3, 4]. However, the data collected in this study described the risk factors in patients with chronic kidney disease in US veterans and not in the general population. Another study by Sung et al. showed that non-apnea sleep disorders might be an early indicator of cognitive dysfunction, especially in the younger population [26], even though those without non-apnea sleep disorders were not included in this study. There was a small group of patients with subjective insomnia (ICD-9-CM codes 307.42, 307.49, and 780.52). This type of insomnia is also known as sleep state misperception. Personality traits, anxiety, rumination, and pre-sleep worry have been suggested to be associated with such cases of insomnia. The somatic preoccupations of these insomniacs are most likely not hypochondriacal in nature but rather reflect true physiological and physical changes that occur as a result of chronic activation of the stress system. The distinct psychological profiles between insomnia subtypes based on objective sleep duration have showed that patients with subjective insomnia have higher neuroticism, higher scores on the psychasthenia and schizophrenia scales, higher anxiety, lower mood, more dysfunctional sleep-related cognitions, and fewer somatic complaints compared to patients with objective insomnia [27]. This type of insomnia is related to stress.

Another mechanism that possibly links primary insomnia to dementia is amyloid β-related neurodegenerative pathophysiological processes [9]. Proteins, including amyloid β and tau, which are linked to neurodegenerative diseases, are present in the interstitial space surrounding brain cells [28]. Cerebrospinal fluid recirculates throughout the brain, interchanges with interstitial fluid, and removes interstitial proteins, including amyloid β. Amyloid β clearance is increased during sleep, and the sleep-wake cycle regulates lymphatic clearance [29]. Primary insomnia therefore leads to an accumulation of amyloid β deposits, potentially triggering earlier cognitive decline [29]. Age-dependent amyloid β deposits are related to Alzheimer’s disease and sleep abnormalities [30]. Other studies have reported that amyloid deposits in the preclinical stage of Alzheimer’s disease are associated with worse sleep quality, but not quantity [31]. These studies support the hypothesis that primary insomnia is a potential risk factor for subsequent dementia.

There were several advantages to our study. Our study was based on a nationwide population-based case-control study. Secondly, sleep disorders can lead to stroke, Alzheimer’s disease, multiple sclerosis, deep vein thrombosis, and poorer hospital outcomes [20–24]. However, most epidemiological studies have focused on obstructive sleep apnea [7, 8]. Sleep disorders can be divided into primary insomnia, insomnia associated with medical or mental diseases, and insomnia associated with the consumption or abuse of substances. It is difficult to establish the impact of primary insomnia on disease development. In our study, we excluded patients with insomnia due to medical disease, mental diseases, the consumption or abuse of substances, and organic lesions. Therefore, the main finding of our study was that primary insomnia was a potential risk factor for subsequent dementia. In addition, we found that the risk of developing dementia associated with sleep disorder was higher in younger patients. Several studies have shown that total sleep time, sleep efficiency, and percentage of slow-wave sleep all significantly decrease with age, thus showing that young adults are not able to obtain enough time for sleep. Changes in sleep patterns earlier in life have a greater impact on health. Most dementia studies have focused on older individuals. However, the age of onset is an important consideration for dementia patients [31]. Savva et al. have shown that the association between the pathological features of Alzheimer’s disease and dementia is stronger in younger persons than in their older counterparts. Age will be an important factor to consider while considering the effects of dementia and developing effective strategies for dealing with this condition [32, 33].

Some important limitations in this study need to be addressed. First, detailed information regarding smoking, alcohol consumption, socioeconomic status, and family history of systemic diseases were not available from the NHIRD. Second, the follow-up period may not have been long enough for patients to develop dementia in our study. Third, the quality of evidence from a nationwide population-based case-control study is lower than that from randomized trials.

## Conclusions

The results of our study supported the hypothesis that patients with primary insomnia have a higher longitudinal risk of developing dementia. Young patients with primary insomnia showed a greater incidence of dementia than older patients. However, very little is known about the type, frequency, or level of insomnia necessary to induce dementia; further research in this area is necessary to confirm our findings.

## Abbreviations

LHID 2000: Longitudinal Health Insurance Database
NHIRD: National Health Institute Research Database
